# The role of sleep disturbance in reduced accuracy on a divided attention task among patients with fibromyalgia

**DOI:** 10.1097/PR9.0000000000001122

**Published:** 2024-01-12

**Authors:** Jenna M. Wilson, Samantha M. Meints, Robert R. Edwards, Jolin B. Yamin, David J. Moore

**Affiliations:** a Department of Anesthesiology, Perioperative and Pain Medicine, Brigham and Women's Hospital, Boston, MA, USA; b School of Natural Sciences and Psychology, Liverpool John Moores University, Liverpool, United Kingdom

**Keywords:** Attention, Executive function, Cognition, Sleep disturbance, Chronic pain

## Abstract

**Introduction:**

Patients with fibromyalgia show impaired cognitive performance compared with healthy, pain-free controls. Sleep disturbance, anxiety, and depression are highly prevalent among patients with fibromyalgia, and each is associated with impaired cognitive performance. Yet, limited work has explored whether psychosocial factors contribute to group differences in cognitive performance.

**Objectives:**

This secondary data analysis investigated differences in cognitive performance between patients with fibromyalgia and healthy controls, and whether psychosocial factors accounted for these differences.

**Methods:**

Adults with fibromyalgia (N = 24) and healthy, pain-free controls (N = 26) completed 2 cognitive tasks and the Patient-Reported Outcomes Measurement Information System sleep disturbance, anxiety, and depression short forms. Independent samples *t* tests were used to test for differences in cognitive performance between patients with fibromyalgia and healthy controls. Pearson correlations were conducted to examine associations between psychosocial factors and cognitive performance. Psychosocial factors significantly related to cognitive performance were explored as potential mediators of group differences in cognitive performance.

**Results:**

Patients with fibromyalgia demonstrated poorer accuracy for divided attention compared with healthy controls, and sleep disturbance mediated this group difference. On the attentional switching task, healthy controls showed a greater switch-cost for accuracy compared with patients with fibromyalgia, but there was no group difference in reaction time. Anxiety and depression were not related to cognitive performance.

**Conclusion:**

We found that patients with fibromyalgia reported greater sleep disturbance and, in turn, had poorer accuracy on the divided attention task. Sleep disturbance is modifiable with behavioral interventions, such as cognitive behavioral therapy, and may be a target for improving sleep quality and cognitive performance among patients with fibromyalgia.

## Abstract 2

Patients with fibromyalgia reported greater sleep disturbance, which contributed to reduced accuracy on a divided attention task compared with healthy controls.

## 1. Introduction

Fibromyalgia, characterized by widespread pain, stiffness, mood disorders, fatigue, and cognitive difficulties,^26^ affects 2% to 4% of the population.^10^ Impairment in attention and executive functioning is commonly observed in patients with fibromyalgia compared with healthy controls.^2,7,19^ Previously, we showed that chronic pain (fibromyalgia) and experimentally induced acute pain differentially affected cognitive performance, suggesting that the factors underlying the effects of pain on cognitive difficulties in acute and chronic states may differ.^18^ Importantly, fibromyalgia patients report that cognitive difficulties have a large impact on quality of life,^1^ making it important to identify factors that contribute to these difficulties.

Psychosocial factors may be particularly relevant for understanding cognitive difficulties. Sleep disturbance, anxiety, and depression are highly prevalent among patients with fibromyalgia,^25^ and each is related to impaired cognitive performance.^9,23,24^ Interestingly, one study demonstrated that poor sleep accounted for the association between pain severity and impaired attention performance among patients with fibromyalgia.^8^ Yet, limited work has explored whether psychosocial factors contribute to differences in cognitive performance that are often observed between patients with fibromyalgia and healthy controls. The present study was a secondary data analysis investigating differences in cognitive performance between patients with fibromyalgia and healthy controls and whether psychosocial factors accounted for these differences.

## 2. Methods

### 2.1. Participants and procedure

Participants were 24 adults with fibromyalgia and 26 healthy, pain-free controls (HC). For details about the full procedure and inclusion criteria, see our previously published manuscript (https://www.researchgate.net/publication/332976441_The_Effect_of_Induced_and_Chronic_Pain_on_Attention).^18^ Participants completed questionnaires, and then instructions were provided for the cognitive performance tasks. Participants completed a practice trial of each task before the experimental versions. Study procedures were approved by Brigham and Women's Hospital's Institutional Review Board.

### 2.2. Cognitive performance

Participants completed 3 tasks based on the Bath TAP battery,^16^ designed and controlled using E-Prime II professional software.^22^ This battery was established because of the relationships between these measures of cognition and pain among healthy adults and those with headache and menstrual pain.^12,15–17^ In the original report,^18^ we investigated the impact of an acute mechanical pain stimulus on cognitive performance; here, we report performance on these cognitive tasks in the absence of externally applied noxious stimulation. All participants completed the cognitive tasks twice (ie, in the presence and absence of pain stimuli), and the order of the testing sessions was randomized. We also previously reported that we did not find a significant difference in attention span (n-back task) between patients with fibromyalgia and HC,^18^ and thus, the present study focuses solely on the attentional switching and divided attention tasks.

#### 2.2.1. Attentional switching

To measure the ability to alternate between 2 tasks, participants saw a single digit number and made 1 of 2 decisions about these numbers based on a task-cue presented before each of 200 trials. For some trials, participants indicated whether the number was higher or lower than 5. On other trials, participants indicated whether the number was odd or even. Typically, when the task remains the same (2 consecutive odd/even trials), participants will perform faster and more accurately than when there is a switch between tasks (odd/even then low/high). This reduction in performance on switch trials is called a “switch-cost.” Outcome variables for this task were the differences in reaction time and accuracy between repeat and switch trials. For both reaction time and accuracy, positive scores reflect faster performance on repeat compared with switch trials, whereas negative scores reflect better performance on repeat compared with switch trials.

#### 2.2.2. Divided attention task

To measure participants' accuracy while processing >1 source of information concurrently (a measure of attention and executive function^6^), participants performed 2 tasks simultaneously. Participants were presented with a chain of numbers in the center of the screen and 2 lines, either vertical or horizontal, at the periphery of the screen. Participants identified when 3 consecutive odd or even digits were presented and when the 2 lines were presented in different orientations (1 vertical and 1 horizontal). A total of 400 displays were presented with 8 number targets and 8 line targets on every set of 80 displays. Number and line targets were never both presented on the same trial. The outcome variable for this task was accuracy.

### 2.3. Psychosocial factors

The Patient-Reported Outcomes Measurement Information System short forms, which have demonstrated good reliability and validity,^3,11^ measured sleep disturbance, anxiety, depression, and pain severity.^4^

### 2.4. Data analysis

As previously reported,^18^ data were checked for normality and outliers, and outlying data were excluded case-wise. Independent samples *t* tests were conducted to test for differences in cognitive performance and psychosocial factors between patients with fibromyalgia and HC. Pearson correlations were conducted to examine associations between psychosocial factors and cognitive performance among the whole sample. Psychosocial factors significantly related to cognitive performance were explored as potential mediators of group differences in cognitive performance. Mediation analysis was conducted using the PROCESS macro with bias-corrected 5000 bootstrapped resamples.^20^ A post hoc power analysis indicated that a sample of 47 participants was sufficient to detect a medium- to large-sized effect (f^2^ = 0.22), assuming power is 0.80 and α = 0.05.

## 3. Results

Participants (N = 50) were an average age of 38 years (SD = 12.6), and 90% were female. Participants were White (82%), African American/Black (8%), Asian (8%), and more than one race (2%). There were no differences in age, sex, or race between patients with fibromyalgia and HC.^18^ Patients with fibromyalgia demonstrated poorer accuracy for divided attention compared with HC (*P* = 0.028; Table 1). Based on raw accuracy scores on the attentional switching task, patients with fibromyalgia performed similarly across repeat and switch trials, whereas HC showed a larger benefit in accuracy from repeat than switch trials. In other words, HC showed a greater switch-cost for accuracy compared with patients with fibromyalgia (*P* = 0.009). There was no difference in switch-cost reaction time.

**Table 1:** Group differences in cognitive performance and psychosocial factors.

	Fibromyalgia	Healthy controls	*P*	t
Cognitive performance				
Switch-cost reaction time*	81.56 ± 87.84	81.14 ± 123.24	0.990	−0.01
Repeat trials	924.58 ± 251.51	776.74 ± 295.48	0.078	−1.80
Switch trials	1006.14 ± 310.99	857.88 ± 373.78	0.156	−1.44
Switch-cost accuracy*	−0.01 ± 0.03	−0.04 ± 0.04	**0.009**	−2.76
Repeat trials	0.95 ± 0.04	0.97 ± 0.04	0.213	1.27
Switch trials	0.94 ± 0.04	0.93 ± 0.06	0.325	−1.00
Divided attention accuracy	0.48 ± 0.21	0.60 ± 0.16	**0.028**	2.27
Psychosocial factors				
Sleep disturbance	11.00 ± 1.80	8.96 ± 2.21	**<0.001**	−3.53
Anxiety	10.17 ± 3.97	4.83 ± 1.12	**<0.001**	−6.25
Depression	9.39 ± 4.85	4.52 ± 1.09	**<0.001**	−4.71
Pain				
Pain severity	4.8 ± 1.8	1.0 ± 1.5	**<0.001**	−8.1

Bolded *P*-values represent a significant difference between patients with fibromyalgia compared with HC (*P* < .05).

* Switch-cost scores were calculated by subtracting scores for the repeat trials from the switch trials. A positive score for switch-cost reaction time represents a greater cost of switching or a longer time to complete switch trials than repeat trials. A negative score for switch-cost accuracy represents a greater cost of switching or less accuracy for switch trials than repeat trials.

Patients with fibromyalgia reported greater sleep disturbance, anxiety, depression, and pain severity compared with HC. Correlations (Table 2) showed that greater sleep disturbance was significantly associated with less accuracy on the divided attention task (*P* = 0.01), but sleep was not related to accuracy or reaction time on the attentional switching task (*P*s > 0.05). Anxiety and depression were not related to cognitive performance on any task (*P*s > 0.05).

**Table 2:** Correlations between psychosocial factors and cognitive performance.

	Switch-cost reaction time	Switch-cost accuracy	Divided attention accuracy
Sleep disturbance	0.10	0.24	−0.37*
Anxiety	0.10	0.20	−0.17
Depression	0.01	0.05	−0.15
Pain severity	−0.20	0.17	−0.23

A positive score for switch cost reaction time represents a greater cost of switching or a longer time to complete switch trials than repeating trials. A negative score for switch cost accuracy represents a greater cost of switching or less accuracy for switch trials than repeating trials.

* *P* < 0.05.

**Figure 1.**
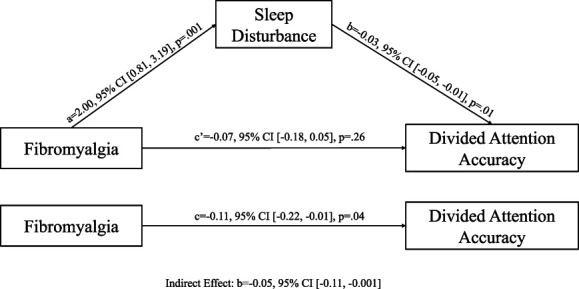
The mediating effect of sleep disturbance in the relationship between fibromyalgia and accuracy on the divided attention task.

We conducted a mediation analysis to explore whether the group difference in accuracy for divided attention performance was mediated by sleep disturbance (Fig. 1). Because sleep was not related to attentional switching, mediation analyses were not conducted for those outcomes. Similarly, as anxiety and depression were not related to cognitive performance, they were not explored as potential mediators.

There was a significant indirect effect of group on divided attention through sleep disturbance (b = −0.05, 95% CI [−0.11, −0.001]). The group difference in divided attention performance was no longer significant when sleep disturbance was included in the model (*P* > 0.05; Fig. 1). This suggests that patients with fibromyalgia demonstrated poorer accuracy for divided attention, and higher sleep disturbance mediated this relationship.

## 4. Discussion

In the present study, patients with fibromyalgia demonstrated poorer accuracy for divided attention compared with healthy controls, which may suggest that patients with fibromyalgia found it difficult to perform this task both quickly and accurately. In the current context where participants performed the timed task with rapidly presented stimuli, it may not have been possible for patients to use compensatory strategies to maintain accuracy. Although we observed a greater switch-cost for accuracy among healthy controls, raw scores on the switching task suggested a slightly larger benefit from repeat trials among healthy controls, whereas patients with fibromyalgia performed similarly across repeat and switch trials.

Research has shown that sleep disturbance is associated with cognitive difficulties, including impaired executive function and attention,^9^ and one study demonstrated that poor sleep mediated the association between pain severity and impaired attentional performance.^8^ In the present study, greater sleep disturbance was associated with poorer accuracy for divided attention. Furthermore, we found that sleep disturbance mediated the group difference in divided attention, such that patients with fibromyalgia reported greater sleep disturbance, and in turn, poorer accuracy for divided attention. Sleep disturbance is a modifiable factor that may be targeted among patients with fibromyalgia to improve cognition. Indeed, randomized controlled trials (RCTs) have shown that cognitive behavioral therapy (CBT) is effective for improving sleep among patients with fibromyalgia.^13,14,21^ Moreover, a meta-analysis found that CBT for insomnia improves sleep quality, while also reducing pain severity, anxiety, and depression,^5^ indicating it may be a beneficial treatment for a variety of comorbid symptoms. Additionally, one study showed that CBT improved executive functioning among patients with fibromyalgia, and that improvements in executive functioning were associated with improved sleep.^14^ More RCTs are needed to examine whether reducing sleep disturbance among patients with fibromyalgia leads to improved cognition, specifically the ability to simultaneously process multiple pieces of information (ie, divided attention).

There are limitations to consider when interpreting our findings. Our sample was majority female and White, limiting the generalizability of our findings. We only included patients with fibromyalgia and the factors that explain cognitive difficulties may differ for other chronic pain conditions. Our measures of cognitive performance mainly assessed aspects of attention and executive function. Future studies should assess other types of cognition, including memory. Despite these limitations, our findings highlight the importance of considering symptoms of sleep disturbance when investigating cognitive performance, particularly executive attention, among patients with fibromyalgia.

## Disclosures

The authors have no conflicts of interest to declare.
